# Application of Immersive and Semi-Immersive Extended Reality Training Systems in Acupuncture Education: Scoping Review

**DOI:** 10.2196/93369

**Published:** 2026-10-21

**Authors:** Hyein Jeong, Hyunsuk Park, Junmoo Ahn, Kyeong Han Kim, Hojung Lee, Taehoon Bang, Min Jun Kang, Sangjun Lee, Youngju Rhee, Seung Kyu Kim, Seong-Gyu Ko

**Affiliations:** 1 Department of Preventive Medicine, College of Korean Medicine, Kyung Hee University, 26 Kyungheedae-ro, Dongdaemun-gu, Seoul, 02447, Republic of Korea, +82 2 961 0329; 2 Department of Korean Medicine, Graduate School, Kyung Hee University, Seoul, 02447, Republic of Korea; 3 Department of Medical Education, College of Medicine, Seoul National University, Seoul, 03080, Republic of Korea; 4 Department of Preventive Medicine, College of Korean Medicine, Woosuk University, Wanju, 55338, Republic of Korea; 5 Department of Applied Korean Medicine, Graduate School, Kyung Hee University, Seoul, 02447, Republic of Korea; 6 Department of Clinical Korean Medicine, Graduate School, Kyung Hee University, Seoul, 02447, Republic of Korea

**Keywords:** acupuncture, education, virtual reality, augmented reality, mixed reality

## Abstract

**Background:**

Acupuncture training requires precise acupoint localization and needle control, yet peer-to-peer practice raises safety concerns and physical phantoms offer limited anatomical fidelity. Extended reality (XR) is increasingly used in health professions education, but immersive and semi-immersive XR training systems for acupuncture have not been mapped.

**Objective:**

This scoping review aimed to map the applications, hardware modalities, and reported technical and educational outcomes of immersive and semi-immersive XR training systems in acupuncture education.

**Methods:**

This review followed the PRISMA-ScR (Preferred Reporting Items for Systematic Reviews and Meta-Analyses extension for Scoping Reviews) and the five-stage scoping review framework of Arksey and O’Malley, as refined by Levac and colleagues. On September 19, 2026, we searched PubMed, Embase, Scopus, IEEE Xplore, CNKI, RISS, ScienceOn, and OASIS. No language or date restrictions were applied. Backward and forward citation searching supplemented the database search. Eligible sources were peer-reviewed original studies or full conference papers describing virtual reality (VR; either fully immersive [IVR] or semi-immersive [SIVR]), augmented reality (AR), or mixed reality (MR) systems for acupuncture training. Two reviewers independently screened records and charted the study design, hardware, training task, and outcomes. Each study was assigned an evidence tier.

**Results:**

Of 6176 records, 3378 were screened after removal of duplicates and ineligible document types, 80 reports were assessed in full text, and 26 studies were included (25 from databases and 1 from citation searching). Most studies came from East Asia (25/26, 96.2%), mainly China (n=18), and 20 (76.9%) were published between 2020 and 2026. Systems used VR (n=15), AR (n=5), or MR (n=6); 19 were IVR and 7 were SIVR. Training tasks addressed acupoint localization (n=13), needling skills (n=11), and clinical reasoning (n=2). Localization errors were reported with different metrics and were not directly comparable across systems. In a randomized comparison, a VR group scored higher on practical skills than a conventional instruction group (32.52, SD 2.28 vs 28.62, SD 2.77 points; *P*<.001). In an MR simulator with depth-responsive vibrotactile feedback active in both sessions, mean depth error decreased from 6.41 mm in the first practice session to 3.58 mm in the second. Of the 26 studies, 7 used a comparative design; the rest were technical validation (n=6), system development (n=10), or case and survey reports (n=3). Reported barriers included headset weight, narrow field of view, tracking instability, and limited tactile fidelity.

**Conclusions:**

Immersive and semi-immersive XR systems for acupuncture training have been developed mainly for acupoint localization and needling. Randomized comparisons reported higher test scores than conventional instruction, but most evidence came from system development reports and small feasibility studies. Randomized trials comparing XR-based and conventional training on objective localization and needling outcomes are needed before these systems are adopted for competency assessment.

## Introduction

Acupuncture is one of the most commonly used interventions for pain management, particularly within traditional Chinese medicine and Korean medicine. It is a therapeutic technique that involves the insertion of fine needles into anatomically defined points on the body [1]. Accurately locating and needling these acupoints is inherently difficult due to the subtle anatomical variations and the precision required in point identification [2,3]. In current educational settings, acupuncture training often relies on instructor demonstration and practice on students’ own bodies [4]. While this hands-on approach provides valuable experiential learning, it also raises safety and ethical concerns, particularly with invasive techniques and high-risk acupoints where complications such as pneumothorax may occur [5]. Although sensory and phantom models have been introduced as alternatives to human participants, recent competency assessments of acupuncture and moxibustion clinical skills in Korean medicine education have noted that anatomical models require further refinement to fully capture procedural competency [6].

Consequently, medical education is shifting toward digital tools like simulations and 3D modeling, a trend significantly accelerated by the COVID-19 pandemic [7,8]. In recent years, immersive extended reality (XR) systems have incorporated features intended to address the limitations of earlier systems. Recent studies have demonstrated augmented reality (AR) systems that overlay 3D acupoint models directly onto the human body using markerless tracking [9], and mixed reality (MR) devices like HoloLens 2 that integrate wearable haptic interfaces to provide real-time tactile feedback during needling [10]. These features allow learners to visualize complex internal structures, which may help bridge the gap between theory and practice.

While interest in applying XR to acupuncture education has increased, a synthesis evaluating its pedagogical implementation remains limited. Existing literature reviews in this domain present clear structural constraints. For instance, Jang et al [3] exclusively evaluated physical phantom models and mechanical simulators, thereby leaving virtual learning environments unexamined. Similarly, broader systematic reviews in health care education, such as Sung et al [11] have confirmed the general educational use of virtual reality (VR). However, these syntheses do not address the specific spatial and psychomotor requirements of acupuncture training, including 3D acupoint localization, dynamic needle depth control, and layer-by-layer anatomical navigation. Recent systematic reviews in medical and surgical education by Qi et al [12] and Barteit et al [13] describe a shift from conventional 2D desktop displays toward head-mounted display (HMD)–based extended reality, reporting that spatial immersion and 1:1 physical mapping were associated with improved spatial awareness and psychomotor skill acquisition.

This gap has practical consequences. As health professional curricula adopt competency-based training, curriculum designers and technology developers need to know which immersive modalities are mature enough for which learning objectives and what barriers. We did not identify a review that systematically mapped this evidence for acupuncture, and prior reviews have treated XR as a single intervention rather than distinguishing immersion levels and hardware modalities by training task. This scoping review, therefore, maps immersive and semi-immersive XR technologies in acupuncture training, focusing on platforms that support high-fidelity spatial interaction and 1:1 physical mapping, and relates each modality to the training task it addresses and the design of the studies that evaluated it. Based on the PCC (population, concept, context) framework, the review addressed the following research questions:

1. What is the range of immersive and semi-immersive XR technologies used in acupuncture education?
2. How are these technologies integrated to achieve specific educational objectives, such as acupoint localization or needling skill mastery?
3. What evidence exists regarding the technical accuracy and educational efficacy of these systems, and what are the primary barriers to their implementation?

## Methods

### Protocol and Registration

This scoping review was conducted in accordance with the PRISMA-ScR (Preferred Reporting Items for Systematic Reviews and Meta-Analyses extension for Scoping Reviews) guidelines (Checklist 1) [14]. The methodological framework was guided by the five-stage process originally defined by Arksey and O’Malley [15] and further refined by Levac et al [16] to ensure a rigorous and transparent evidence synthesis. To enhance research integrity and prevent duplication, the study protocol was prospectively registered on the Open Science Framework [17].

### Eligibility Criteria

Eligibility criteria were defined using the PCC framework. The Population encompassed learners and practitioners at various stages of professional acupuncture training, including undergraduate and graduate students, clinical interns, trainees, and practicing experts (eg, licensed acupuncturists or traditional medicine doctors). Studies were included if the target population involved individuals acquiring, developing, or evaluating acupuncture skills within institutional or experimental training settings. Regarding the concept, this review categorized XR systems into nonimmersive VR (NIVR), semi-immersive VR (SIVR), and fully immersive VR (IVR) levels using a study-specific operational classification based on display modality, spatial registration, and interaction characteristics, adapted from the classical taxonomy of Ware et al [18] and Lombard and Ditton [19], as summarized in a review of VR research by Cipresso et al [20]. We included IVR (eg, HMD-based systems) and SIVR (eg, 3D stereoscopic displays and UHD touch tables) that achieved 1:1 spatial mapping, defined as true-scale correspondence between the tracked position of the learner’s head, hand, or needle, or the registered position of a real body or physical model, and the position of the virtual content. IVR required an HMD with head tracking and 3D input through tracked controllers, data gloves, hand tracking, or a tracked needle device. SIVR required a display that did not meet the IVR criteria, such as a life-size, stereoscopic, or handheld screen or a monocular head-worn display, with virtual content presented at true scale and either registered to a real body or model or manipulated with tracked, sensor-based, or haptic input; registration and direct manipulation were treated as alternative, not jointly required, routes to 1:1 spatial mapping. Systems operated only by mouse, keyboard, or touch on a desktop monitor or reduced-scale screen without registration to a body, 360 degree video without manipulation, and reports that did not describe the display device were classified as NIVR and excluded, unless the authors stated that learners practiced in a VR system, in which case immersion was assigned from the system description. The context was acupuncture training, including acupoint localization, needling skills, and acupuncture-related clinical reasoning. Inclusion was limited to peer-reviewed original research and full conference papers; no language restriction was applied. Theses, reviews, editorials, study protocols, trial registry records, book chapters, preprints, and conference abstracts were excluded.

### Information Sources

We searched eight electronic databases: PubMed (National Library of Medicine interface), Embase, Scopus, IEEE Xplore, CNKI (overseas platform), RISS, ScienceOn, and OASIS. All database searches were run on September 19, 2026. Backward and forward citation searching of the included studies supplemented the database search.

### Search

The search was developed and is reported in accordance with PRISMA-S (Preferred Reporting Items for Systematic Reviews and Meta-Analyses Literature Search Extension) guidelines (Checklist 2) [21]. Search strategies combined a context block (acupuncture, acupoints, meridians, and East Asian medicine terms) with a concept block (VR, AR, MR, XR, haptic, HMD, and related device terms) using the Boolean operator AND. The population element and outcome terms were applied at the eligibility stage rather than in the search, because restricting searches by additional concepts reduces sensitivity [22]. Controlled vocabulary (MeSH in PubMed and Emtree in Embase) was combined with free-text terms in title, abstract, and keyword fields. No published search filters were used or adapted, and no search strategy from a previous review was reused.

Syntax was adapted to each database. IEEE Xplore limits a query to 25 terms, so the concept block was reduced to 13 core terms. OASIS indexes only Korean medicine literature, so the concept block was run alone. In CNKI, the subject field was searched using Chinese acupuncture and XR terms. No language, date, or study design limits were applied. The search strategies were internally reviewed by the review team before the searches were run, and the searches were not subsequently rerun or updated. The search strategy for each database, copied exactly as run, with the number of records retrieved at each step, is provided in Multimedia Appendix 1.

### Selection of Sources of Evidence

Records were exported from each database and deduplicated in Microsoft Excel by matching digital object identifiers and normalized titles within a publication-year window of 2 years. Screening then proceeded in 2 stages.

At the title and abstract stage, 2 reviewers (HIJ and HSP) independently screened all records, without access to each other’s judgments, against the predefined eligibility criteria, and disagreements were resolved by discussion. Records without an abstract were judged on the title, and abstracts were retrieved from the source database when the title was insufficient.

At the full-text stage, the same 2 reviewers independently assessed each report against the eligibility criteria, and disagreements were resolved by discussion. Exclusions were coded as nonimmersive system (F1), no educational purpose (F2), no implemented system or outcome, or a conference abstract (F3), ineligible publication type (F4), or full text not retrievable (F5).

### Data Charting Process

Data charting was performed independently and in duplicate by 2 reviewers (HIJ and HSP) using a standardized charting form; the variables charted are listed under the *Data Items* section. The charting form was pilot-tested on a subset of included sources before full charting to calibrate its application. The completed forms were compared item by item, and disagreements were resolved by a third reviewer (KHK).

### Data Items

For each included source, the following data items were charted: author, year of publication, country, XR modality and level of immersion, study design, participants and sample size, training content and task, reported outcomes and metrics, hardware and software, and limitations. Country was assigned according to the corresponding author’s affiliation, and Hong Kong was counted within China.

### Critical Appraisal of Individual Sources of Evidence

Consistent with established scoping review methodology, formal critical appraisal of the methodological quality or risk of bias of individual sources of evidence was not performed, as the objective of this review was to map the extent, range, and nature of the available evidence rather than to evaluate its quality; this scope decision is discussed further in the *Limitations* section.

### Synthesis of Results

Charted data were summarized descriptively and narratively. Study characteristics were tabulated and reported as frequencies and percentages by country, year of publication, and level of immersion. Included studies were then grouped by the core educational task they targeted, namely acupoint localization, needling skills, and clinical reasoning, and the reported technical and educational outcomes were summarized narratively within each group. Each study was assigned to one domain according to the principal training objective stated by the authors, and outcomes for other tasks were described narratively. To describe the study designs underlying the available evidence, each source was additionally assigned to 1 of 4 evidence tiers: tier 1 for comparative or experimental designs; tier 2 for technical validation; tier 3 for system or hardware development; and tier 4 for case, survey, or qualitative reports, and the distribution of these tiers across modalities and educational domains was displayed as an evidence gap map. These tiers reflect study design categories used for descriptive mapping and are not a validated hierarchy of methodological quality or risk of bias. Finally, identified limitations and future directions for formal curriculum integration were synthesized.

## Results

### Selection of Sources of Evidence

Figure 1 illustrates the study selection process. The database searches identified 6176 records (PubMed, n=548; Embase, n=1576; Scopus, n=1456; IEEE Xplore, n=798; CNKI, n=855; RISS, n=450; ScienceOn, n=476; OASIS, n=17). Before screening, 1171 records of ineligible document types were removed through database filters, including 106 Embase trial registry records, and 1627 duplicates were removed. The remaining 3378 records were screened by title and abstract, and 3291 were excluded. Of these, 260 addressed treatment in patients or self-care use and 2288 were unrelated to acupuncture education. A further 99 described no XR training system, 642 were ineligible publication types, and 2 were duplicates identified during screening.

**Figure 1.**
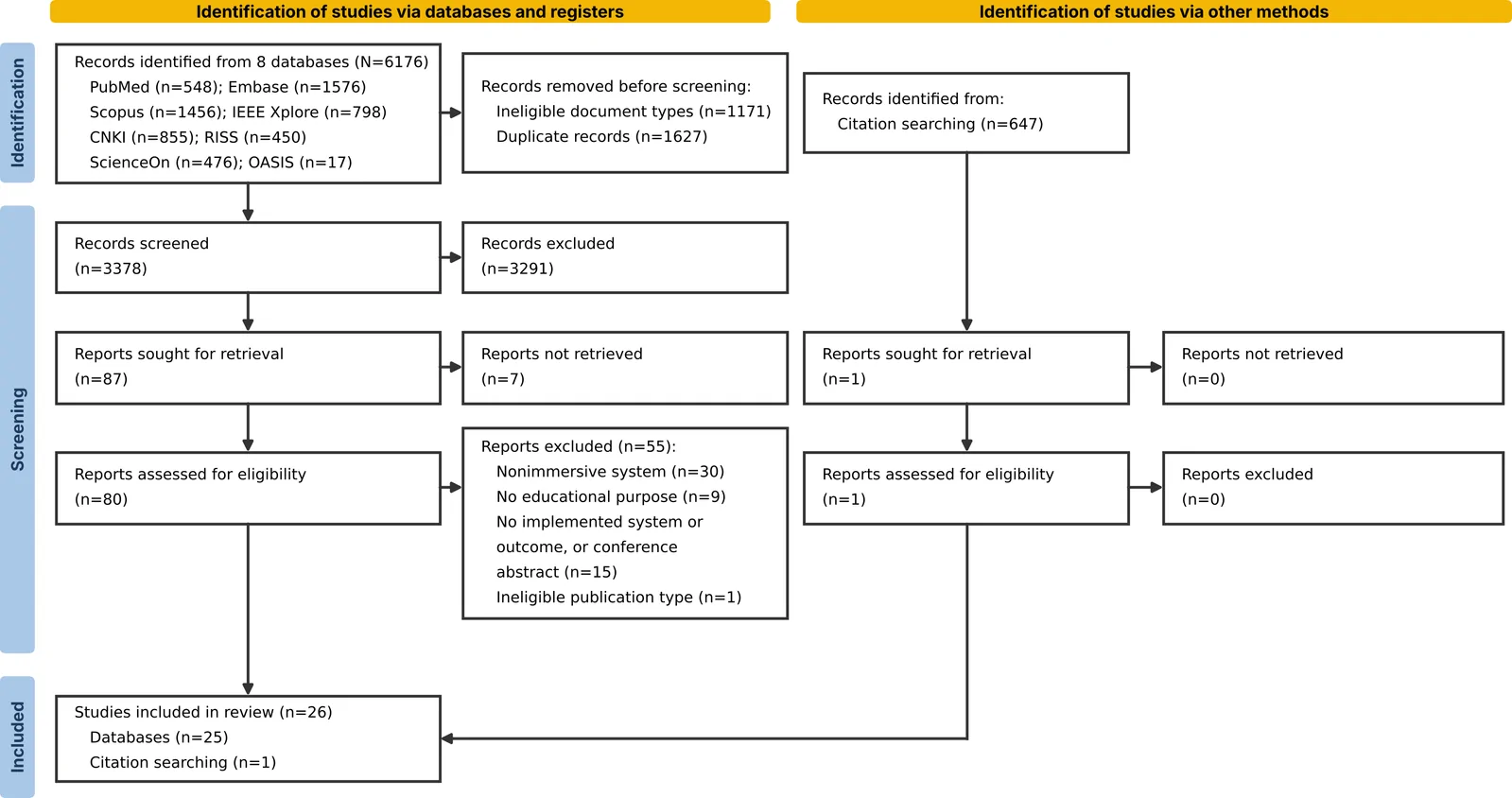
PRISMA-ScR (Preferred Reporting Items for Systematic Reviews and Meta-Analyses extension for Scoping Reviews) flow diagram of the study selection process.

Of the 87 reports sought for retrieval, 7 could not be obtained. Of the 80 reports assessed in full text, 55 were excluded. Reasons were a nonimmersive system (n=30), no educational purpose (n=9), no implemented system or outcome, or a conference abstract (n=15), and an ineligible publication type (n=1).

Citation searching identified 647 records. These records were screened by title and abstract against the database search results and the same eligibility criteria; 646 were excluded because they duplicated a database record or did not describe an XR training system for acupuncture, and the remaining record was assessed in full text and included [23].

In total, 26 studies were included in this scoping review, 25 from the database search and 1 from citation searching (Figure 1).

### Characteristics of Sources of Evidence: Geographical and Temporal Distribution

A geographical analysis of the 26 included studies showed that research conducted in the East Asian region accounted for 96.2% (n=25) of the total. By country, China produced the highest number of studies with 18 publications [4,23-39], followed by South Korea with 4 [10,40-42] and Taiwan with 2 [9,43]. A single study was reported from the United States [44], and one from Japan [45] (Figure 2). Six studies were published before 2020, in 2008 [45], 2013 [37], 2015 [44], 2017 [39], and 2019 [26,35], and the remaining 20 studies (76.9%) were published between 2020 and 2026 (Figure 3) [4,9,10,23-25,27-34,36,38,40-43].

**Figure 2.**
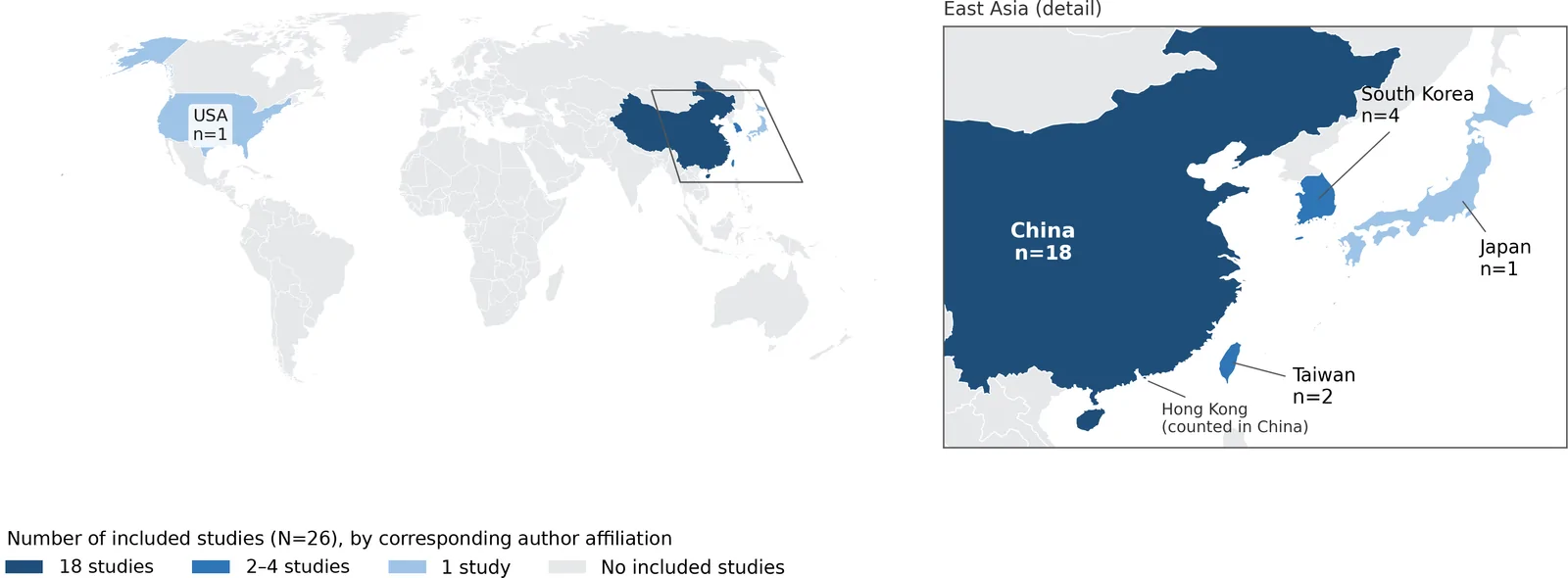
Geographical distribution of the 26 included studies by country of the corresponding author’s affiliation. The inset shows East Asia in detail; Hong Kong was counted within China.

**Figure 3.**
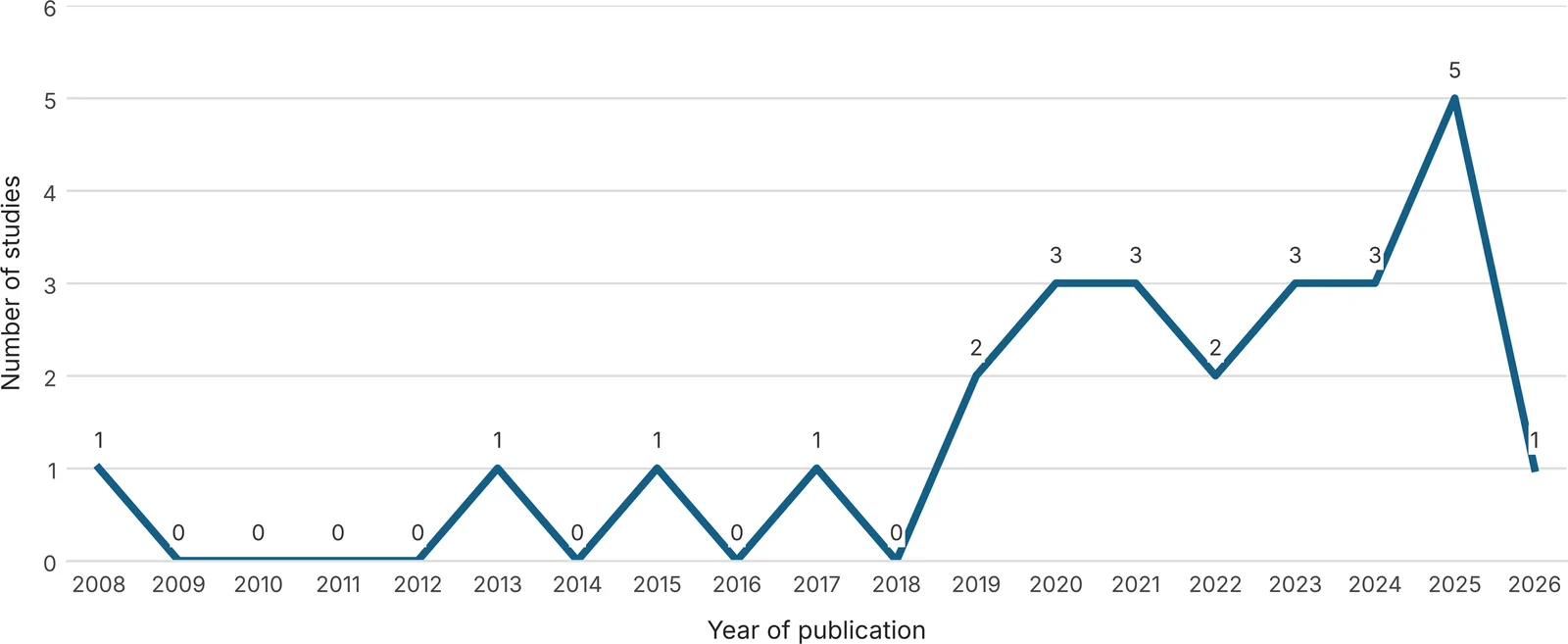
Number of included studies by year of publication.

### Classification of Immersive and Semi-Immersive XR Systems

XR systems can be distinguished into 3 types based on the level of immersion experienced by the user: NIVR, SIVR, and IVR [18-20]. This review focused on immersive and semi-immersive technologies that meet the criteria of high-fidelity spatial interaction and 1:1 physical mapping. Fully immersive systems (IVR, n=19), which use HMDs and 3D input devices (eg, VR controllers or data gloves) or markerless bare-hand pinch interactions with hand-tracking algorithms (eg, HoloLens 2) to completely immerse the user in the virtual environment, represented the majority of the included studies [9,10,23-27,30-35,37-41,45]. Semi-immersive systems (SIVR, n=7) typically consist of large screens for projection and advanced interface devices (eg, haptic feedback or infrared cameras) that allow users to interact with the immersive environment while remaining aware of the physical world, providing partial immersion and high presence [18-20]. This category included a life-size interactive touch table for which an AR module was planned but was not yet implemented [42], large-screen projection combined with student practice in a VR system [4], a stereoscopic display with 3D glasses and an interactive stylus [28], and a VR system described with stereoscopic glasses or a headset [29]. Other semi-immersive systems used a tracked life-size physical model with a large display [36], a tablet interface registered 1:1 to the head [43], or a monocular head-worn display (Google Glass) registered to the face through its camera [44]. By modality, 15 studies used VR, 5 used AR, and 6 used MR.

### Results of Individual Sources of Evidence: Analysis of Key Training Tasks and Learning Outcomes

Educational tasks were categorized into 3 domains: acupoint localization (n=13), needling skills (n=11), and clinical reasoning (n=2).

First, acupoint localization (n=13) involved learning acupoint locations on 3D virtual bodies or by overlaying virtual data onto a real body or physical model. Regarding technical precision, Chen et al [27] reported a mean positional error of 2.55 mm for 16 facial acupoints overlaid through HoloLens 2 on 8 volunteers. Chiou et al [9] reported a mean error of 5.01 (SD 2.64) mm using a “Six-Point Landmark-Based AR Registration” algorithm that maps acupoints directly onto the patient’s body without external markers, which the authors considered clinically acceptable. Several systems allowed learners to view the 3D relationship between acupoints and underlying structures such as muscles, bones, nerves, and vessels [4,25,28,42]. In the study by Sun et al [30], an MR group scored higher than a textbook group on point-location items of a knowledge test given twice, 1 week apart (*P*=.03). A tablet-based AR system with 6-point registration showed discrepancies of 0.6 to 3.9 mm between virtual and actual acupoints on a mannequin head, with larger depth errors along the axis perpendicular to the screen [43]. These values are not directly comparable because the studies measured error of facial acupoint estimation [27], registration error on the body [9], and virtual-to-actual discrepancy on a tablet screen [43]. One system identified acupoints with a tracked pointer on a life-size copper model [36], and another supported acupoint selection in a head-mounted VR environment [35]; neither reported learner outcomes. Two further head-mounted VR systems let learners explore meridians and acupoints on a 3D body with hand controllers [25,26]. One reported only informal feedback from teachers and students [25], and the other reported no user data [26]. Two comparative studies assessed localization skills. In a randomized comparison of 70 vocational students, a VR group scored higher than a conventional group on acupoint localization (90.26, SD 3.11 vs 84.12, SD 3.98 points; *P*<.001) [29]. Compared with earlier student cohorts (98 VR-group vs 122 earlier-cohort students), a stereoscopic VR system was associated with higher practical examination scores, of which acupoint localization made up 70% (88.66, SD 6.82 vs 86.59, SD 8.41 points; *P*<.05) [28]. A wearable AR system on Google Glass mapped personalized acupoints and recorded complete treatment sessions, with an accuracy bias of 7.7 to 8.6 pixels against an electronic acupoint detector in three volunteers [44]. In a survey of 180 students using a head-mounted VR acupuncture system, 86% reported liking the system and 89% reported greater interest [34].

Second, needling skills (n=11) focused on training for precise control of needle depth, angle, and stimulation intensity. Recent studies have evolved beyond simple visual cues to enhance skills through “sensory feedback” and real-time collision detection. In the study by Guruge et al [10], vibrotactile feedback that intensified with insertion depth was active in both practice sessions, and mean depth error decreased from 6.41 mm in the first session to 3.58 mm in the second. Experts scored higher than novices (69.25, SD 21.64 vs 56.26, SD 23.37 points), which the authors interpreted as support for construct validity. Yan et al [24] integrated a haptic device that replicated tissue resistance while learners needled back acupoints selected from case sheets; no learner evaluation was reported. Sun et al [30] implemented a multilayer collider algorithm to gauge insertion depth and angle in real time; across 2 trials, task time and the number of mistakes decreased, whereas angle and depth accuracy did not change significantly (*P*=.41 and *P*=.06). Early research by Kanehira et al [45] combined a head-mounted display with a magnetically tracked training device to judge the position, depth, and angle of a virtual needle in real time, without participant testing. Xu and Li [4] described a teaching reform in which an instructor’s headset view of high-risk acupoints was projected to the class before students practiced needling in a VR system with automatic assessment; no comparison group was reported. Most recently, a randomized comparison by Wu et al [31] found that an IVR system (PainEaseVR) was associated with higher posttraining practical-skills scores than conventional instruction (mean 32.52, SD 2.28 vs mean 28.62, SD 2.77 on an 18-item checklist; *t*_58_=5.947; *P*<.001). In a randomized comparison of 70 students, a group taught in a shared head-mounted VR scene outscored a conventional group [32]. Scores were 86.84, SD 5.04 vs 78.26, SD 7.11 points for theory and 91.37, SD 3.16 vs 85.23, SD 4.21 points for operational skills (both *P*<.001). An MR system that combined ultrasonic computed tomography with HoloLens 2 guidance was tested with 31 participants [38]. It received higher System Usability Scale scores and lower NASA Task Load Index mental demand than a two-ring interface. Hand input was also captured with a data glove and 3D tracker, tested informally with 12 students [37], or with machine learning gesture recognition that reached about 92.65% average accuracy in the VR application [39].

Third, clinical reasoning (n=2) evaluated integrated decision-making, such as syndrome differentiation and treatment selection, through case-based scenarios. According to Jiang et al [23], the group taught with VR achieved a mean case analysis score of 93.88 (SD 4.20) compared with 84.65 (SD 4.68) in the traditional group (*P*=.001). In a survey of 79 students after an HTC Vive Pro acupuncture anesthesia scenario, 99% reported that the experience helped their clinical thinking [33].

### Hardware Environment and Implementation Barriers

The reviewed studies utilized a wide range of hardware to construct educational systems, from high-performance HMDs (HTC Vive, HoloLens 2, and Magic Leap One) to tablets, stereoscopic monitors, and specialized UHD multitouch tables (Table 1). To enhance tactile realism or optimize rendering performance on standalone devices, one study integrated a custom ESP32-based wearable haptic unit [10], and another applied polygon retopology to complex 3D anatomical models [30]. Despite these advancements, several barriers to implementation in actual educational settings were identified.

**Table 1.** Summary of included studies on extended reality (XR) technologies for acupuncture education.

Author (year) and country	XR type	Design and participants (N)	Training content and task	Detailed outcomes and learning metrics	Core hardware	Key limitations
Lu (2021), China [25]	VR^a^ (IVR^b^)	Technical development and pilot study (N not reported); teachers and students	Controller-based exploration of the 12 meridians and acupoints with muscles, bones, and organs	Polygon count 130 k→20 k; informal teacher and student feedback: generally satisfied	VR headset and hand controllers (model not reported); UE4^c^	Physical movement restricted by hardware cables; issues with SDE^d^
Jiang (2024), China [23]	VR (IVR)	Randomized controlled trial (n=50); TCM^e^ clinical internship students	Case-based pathogenesis, syndrome differentiation, and treatment (including acupuncture) for internal medicine	Theory 94.25 (SD 5.10) vs 85.36 (SD 5.02); case analysis 93.88 (SD 4.20) vs 84.65 (SD 4.68) (both *P*=.001); satisfaction 96.0% vs 72.0% (*P*<.05)	VR headset (model not reported)	Cannot fully replace high-fidelity clinical patient interactions
Yan (2024), China [24]	VR (IVR)	System design; needs analysis survey and interviews only; no learner evaluation	Theory mode and case-based needling mode: learners read a case sheet and needle the matching back acupoint with force feedback	Real-time haptic force feedback (OpenHaptics) simulating tissue resistance	HTC VIVE headset; Touch haptic interface device	Current technology struggles to perfectly replicate subtle clinical Deqi sensations
Chen (2025), China [29]	VR (SIVR)^f^	Randomized controlled trial (n=70); vocational college students	Acupoint identification and localization module (evaluated module)	Theory 85.27 (SD 4.83) vs 77.34 (SD 5.87); acupoint localization skill 90.26 (SD 3.11) vs 84.12 (SD 3.98) (both *P*<.001)	VR system (device for evaluated module not reported; system description names 3D glasses or VR headset)	Real-time GPU rendering and system stability significantly impact learner immersion
Chiou (2025), Taiwan [9]	AR (IVR)	Technical validation study (n=10); students and human participants	Navigation and identification of 8 major cranial/facial acupoints using markerless registration	Positional error 5.01 (SD 2.64) mm (95% CI 4.27‐5.76); in stability testing, drift SD below 2 mm for 87.5% of measurements	Magic Leap One AR^g^ Glasses	High procurement cost; potential scanning interference from human hair
Guruge (2025), South Korea [10]	MR^h^ (IVR)	Construct validity and usability study, two sessions, no control group (n=10); 8 novices and 2 experts	Depth-controlled needling practice (shallow/medium/deep) with vibration feedback	Depth error 6.41→3.58 mm between sessions (haptics on in both); experts >novices (69.25 [SD 21.64] vs 56.26 [SD 23.37]), construct validity	Microsoft HoloLens 2, ESP32 Haptic Device	Minor tracking latency issues; does not simulate actual tissue resistance
Ryu (2020), South Korea [40]	MR (IVR)	System performance tests (50 trials); no learner evaluation	Comprehensive learning of 361 standard acupoints based on WHO^i^ regulations	94.0% manikin-virtual matching accuracy; 76.59 fps rendering	HTC VIVE Pro, Standardized Dummy	Needs further integration of specialized, high-precision needling controllers
Ryu (2020), South Korea [41]	MR (IVR)	Hardware design and performance tests; no learner evaluation	Design and evaluation of needling controller and full-body manikin for objective training	96% matching; 100% motion recognition	Custom controller with pressure and position sensors; VIVE Pro	Primary focus on hardware stability; no learner data
Yang (2021), South Korea [42]	AR (SIVR)	Development report; prototype with AR module not yet implemented; no user evaluation	Integrated platform for acupoint localization and OSCE^j^ evaluation using multilayered models	Layers 0‐2 (acupoints, skin, and muscle) of a planned multilayer model implemented; no outcome data	KM^k^ SMART Table (UHD Display)	High initial setup costs; limited haptic interaction compared to wearable HMDs^l^
Xu (2025), China [4]	VR (SIVR; student device not reported)	Case study and educational reform analysis; BUCM^m^ students	Instructor headset view projected to class, then student VR practice with automatic assessment on anatomy and needling of high-risk acupoints	Questionnaire: VR useful for memorizing and understanding; learning-time data from 180 users in 2023	Instructor VR headset with large-screen projection; student device not reported	Lacks large-scale quantitative statistical metrics for academic comparison
Gao (2019), China [26]	VR (IVR)	Development report; no user evaluation	JSON-based data management and ray-casting interaction via controllers	Key technologies implemented; no outcome data	HTC VIVE Platform	Internal anatomical structures (muscles/bones) not fully detailed in current model
Kanehira (2008), Japan [45]	VR (IVR)	System development; no participant data	Virtual needle driven by a tracked training device; real-time judgment of sting-point position, depth, and angle on a 3D arm model	Quantitative depth indexing; real-time pass/fail judgment	HMD with Polhemus magnetic sensors on head and hand; tracked training device	Early research stage; lacked force feedback and velocity indexing
Jiang (2015), the United States [44]	AR (SIVR)	System development and feasibility test (n=6 volunteers; accuracy test in 3)	Personalized acupoint mapping and treatment session recording/quantification	Accuracy bias 7.7‐8.6 px against an electronic acupoint detector (3 volunteers); full-session recording for self-guided learning	Google Glass Wearable	Slight latency due to limited computing power
Chen (2021), China [27]	MR (IVR)	Technical validation and accuracy study (n=8 volunteers)	Real-time face recognition and acupoint overlay using Dlib facial landmarks	Positional error approximately 2.55 mm; 20 fps real-time processing	Microsoft HoloLens 2	Performance is highly sensitive to ambient lighting and physical occlusion
Rao (2020) China [28]	VR (SIVR)	Historical-control comparison (n=220; 98 VR vs 122 earlier cohorts); TCM undergraduates	Meridian and acupoint localization and needling technique on a virtual bronze figure; practical exam 70% localization, 30% needling	Practical exam 88.66 (SD 6.82) versus 86.59 (SD 8.41)(*P*<.05); VR-group satisfaction >90% for interest and interactivity, 84.7% ease of use, 80.6% participation	Stereoscopic display with 3D glasses and interactive stylus with force feedback	Special glasses with limited field of view; one learner per workstation
Sun (2023), China [30]	MR (IVR)	Randomized controlled trial (n=20); medical school students	Direct pinch-based virtual needle insertion using bare hands onto a full-scale 3D virtual human anatomy model	Higher point-location scores than textbook group (F[1,37]=5.165, *P*=.03)^n^; task time and mistakes decreased across two trials (*P*=.002 and *P*=.005)	Microsoft HoloLens 2, MRTK^o^, Unity3D	Narrow field of view and peripheral perspective distortion in the lower workspace impede lower-limb tracking; lacks variable individual anatomical variations and realistic needle penetration force feedback
Wu (2026), China [31]	VR (IVR)	Randomized posttest-only comparative study (n=60); TCM undergraduate students	Self-directed VR training on acupoint distribution, standardized needling operation, and case-based diagnostic scenarios, using controller-based needle insertion with real-time collision-detection feedback	Higher theoretical knowledge (*M*=10.08 [SD 0.99] vs 9.04 [SD 1.31], t[58]=3.484, *P*<.001) and practical skills scores (*M*=32.52 [SD 2.28] vs 28.62 [SD 2.77], t[58]=5.947, *P*<.001) than traditional instruction; Cohen *d*=0.90 (95% CI 0.36‐1.43) for both outcomes^n^	VR headset and controllers; custom platform (PainEaseVR, no formal version number)	Single-institution, posttest-only design (no baseline); 2-week intervention with outcomes assessed immediately post-training (no retention follow-up); no haptic tissue-resistance feedback; VR and control arms differed in feedback source (automated vs instructor-led), confounding the isolated effect of VR
Sun (2023), China [32]	VR (IVR)	Randomized controlled trial (n=70); undergraduate medical students	Acupuncture, tuina and cosmetology practical operations: shared-scene teacher demonstration, then self-directed practice with automatic error prompts	Theory 86.84 (SD 5.04) vs 78.26 (SD 7.11); operation 91.37 (SD 3.16) vs 85.23 (SD 4.21) (both *P*<.001); higher CTDI-CV^p^, problem-solving and satisfaction scores (*P*<.05)	VR head-mounted display (model not reported), cloud course database	HMD model and needling fidelity not described; acupuncture content mixed with cosmetology; single center
Chen (2022), China [33]	VR (IVR)	Cross-sectional post-experience survey (n=79); acupuncture-tuina and other-major students	Acupuncture anesthesia case for thyroidectomy (Futu, Hegu, Neiguan; electroacupuncture) in a virtual operating-room scenario	92% satisfied; 99% reported improved clinical thinking; 97% reported greater interest	HTC Vive Pro headset and data gloves, Unity3D	No control group or objective outcome; weak realism of hand needling without force feedback; single case scenario
Zhang (2022), China [34]	VR (IVR)	Descriptive system report with student survey (n=180); acupuncture students	3D panoramic meridian and acupoint viewing with sectional anatomy, plus gamified acupoint-location and needling app	86% liked the system; 89% reported higher interest; 82% reported improved thinking	Headsets shown in a laboratory photograph (model not reported), mobile quiz app	Survey only; headset model, tracking, and input not described; no objective learning outcome
Geng (2019), China [35]	VR (IVR)	System development; not tested with learners	Identification of acupoints and simulated needling by controller ray selection on a virtual body	Functional implementation reported; no learner outcome	HTC VIVE headset and controllers, Unity3D, SteamVR/VRTK	No evaluation; needling reduced to point selection without depth or angle
Tong (2023), Hong Kong and China [36]	AR (SIVR)	System development; not tested with learners	Life-size (1750 mm) 3D-printed copper acupuncture model on a rotating table; tracked pointer identifies acupoints with beginner and expert modes	Real-time correct or incorrect acupoint identification via tracker-to-model registration; no learner outcome	Two trackers, large display monitor, touch-screen monitor, 3D-printed physical model	No user study; screen-based AR view separate from the model
Chen (2013), China [37]	VR (IVR)	System development; informal test with 12 students	Point finding, needle grasping, insertion and manipulation with a tracked virtual hand and gesture control	Qualitative usability judged acceptable by 12 students; no quantitative outcome	5DT data glove, Flock of Birds 3D tracker, helmet-mounted display or LCD monitor (display used in testing not reported)	No evaluation; limited gesture set; no force feedback
Chiou (2024), Taiwan [43]	AR (SIVR)	Technical validation on a mannequin head	Tablet-based AR navigation of head acupoints using 6-point registration to the recipient	Virtual-to-actual acupoint discrepancy 0.6 to 3.9 mm; error lower near screen center and camera	Tablet camera and screen, 6-point landmark registration	Depth error along the screen normal; manual alignment required; head region only
Wang (2025), China [38]	MR (IVR)	Within-subject comparative user study (n=31) plus formative study (n=13); Medical students, a novice practitioner and expert acupuncturists	Ultrasonic computed tomography-guided needle insertion with MR trajectory display, compared with a two-ring 3DUI^q^ and the finger-cun method	Higher SUS^r^ than the two-ring 3DUI; lower NASA-TLX^s^ mental demand; improved insertion consistency	Microsoft HoloLens 2, ultrasonic CT, NDI optical tracking	Requires UCT^t^ scanning and marker tracking; tested on the arm region only; small expert sample
Zhang (2017), China [39]	VR (IVR)	Technical validation study (N not reported)	Recognition of acupuncture hand gestures for virtual needle manipulation	BPNN^u^ 96.98% (training); about 92.65% in the VR application	Oculus headset, Leap Motion	Occlusion limits hand tracking; no learner outcome

a VR: virtual reality.

b IVR: immersive virtual reality.

c UE4: Unreal Engine 4.

d SDE: screen door effect.

e TCM: traditional Chinese medicine.

f SIVR: semi-immersive virtual reality.

g AR: augmented reality.

h MR: mixed reality.

i WHO: World Health Organization.

j OSCE: Objective Structured Clinical Examination.

k KM: Korean medicine.

l HMD: head-mounted display.

m BUCM: Beijing University of Chinese Medicine.

n Values as printed in the original report; in Sun et al [30] the degrees of freedom do not match the reported sample size, and Wu et al [31] reported the same effect size for both outcomes. Key limitations were taken from the study reports or identified by the reviewers from the reported system characteristics.

o MRTK: Mixed Reality Toolkit.

p CTDI-CV: Critical Thinking Disposition Inventory–Chinese Version.

q 3DUI: 3D User Interface.

r SUS: System Usability Scale.

s NASA-TLX: NASA Task Load Index.

t UCT: ultrasonic computed tomography.

u BPNN: back-propagation neural network.

First, regarding physical constraints, learners reported that headsets were heavy or uncomfortable during prolonged use [25,33], and the stereoscopic glasses in one system limited the field of view and allowed only 1 learner per workstation [28]. Furthermore, optical and tracking constraints in standalone headsets, including a narrow field of view, limited display space, and reduced tracking precision during low-range spatial interactions, were noted as potential obstacles to continuous needle manipulation and spatial orientation [30]. Some virtual models also lacked internal anatomical detail, such as muscles and bones [26]. Second, concerning technical limitations, most current haptic and sensory feedback mechanisms, which primarily rely on vibrotactile cues or simple collision algorithms, fall short of replicating the complex physical resistance experienced during actual tissue penetration, thereby limiting the realistic simulation of the deqi sensation [10,30]. Third, the high cost of acquiring 3D scanners and AR glasses was reported as a barrier to large-scale institutional adoption [9]. Other reported constraints were specific to individual systems. In a VR acupuncture anesthesia project, students reported weak realism of hand needling without force feedback, and only one clinical case was available [33]. Tablet-based AR required manual alignment and showed depth error along the screen axis [43], and the ultrasonic computed tomography system required prior scanning and optical marker tracking [38].

To overcome these barriers, future research directions emphasize improving portability by offloading computation to a paired smartphone [44], incorporating realistic tissue resistance gradients or pseudo haptic feedback beyond simple vibration [10,30], and developing data-driven algorithms that dynamically adjust for individual anatomical variations such as body mass and gender differences in acupoint mapping [30]. Furthermore, developing level-based customized programs [4] and professional validation by expert groups [42] were proposed as next steps for XR-based acupuncture education. The distribution of research and the XR technologies applied across each educational domain are visualized in the evidence gap map (Figure 4).

**Figure 4.**
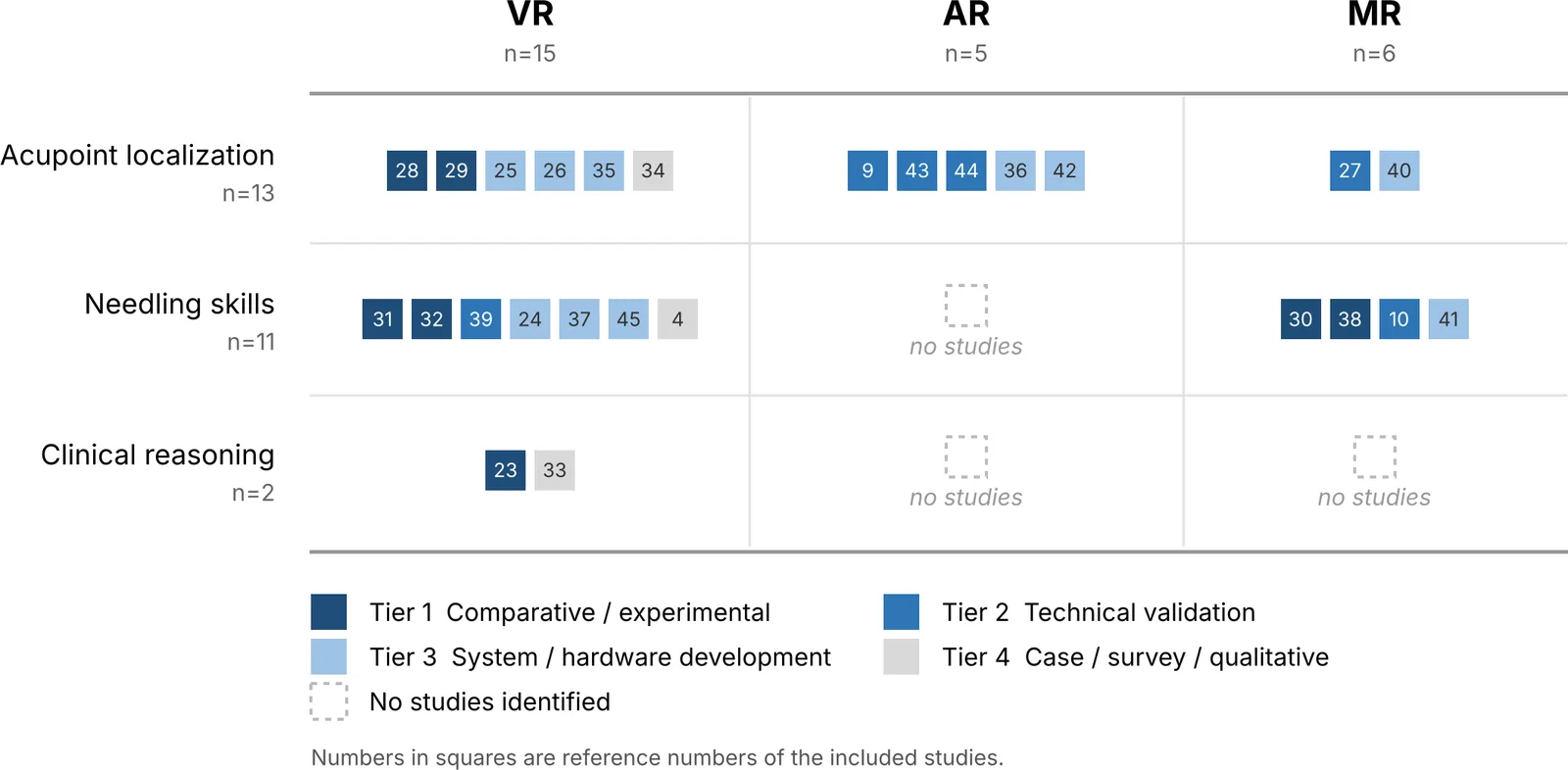
Evidence gap map of the included studies across extended reality (XR) modalities and educational domains. Each square represents 1 included study, colored by evidence tier: tier 1, comparative or experimental design; tier 2, technical validation; tier 3, system or hardware development; tier 4, case, survey, or qualitative report. Dashed outlines mark modality-domain combinations for which no studies were identified [4,9,10,23-45]. AR: augmented reality; MR: mixed reality; VR: virtual reality.

### Synthesis of Results

The distribution of XR modalities differed by domain. As shown in the evidence gap map (Figure 4), AR research was confined to acupoint localization (n=5), and none of the 5 studies used a comparative design. MR studies centered on needling (4 of 6), whereas VR studies addressed localization (n=6), needling (n=7), and clinical reasoning (n=2). Both technologies produced comparative (tier 1) evidence for needling skill training, in VR studies [31,32] and MR studies [30,38], whereas no AR study addressed needling. Among studies assigned to localization, comparative evidence came only from VR studies [28,29], although an MR needling study also compared point-location scores with textbook learning [30]. Clinical reasoning was examined only within VR [23,33]. Overall, 7 of the 26 included studies used a comparative or experimental design (tier 1). The remaining 19 studies comprised technical validation (tier 2, n=6), system or hardware development (tier 3, n=10), and case, survey, or qualitative reports (tier 4, n=3).

These results address the 3 research questions on the range of XR technologies, their educational objectives, and the evidence on technical accuracy, educational efficacy, and implementation barriers.

## Discussion

### Summary of Evidence

This scoping review mapped immersive and semi-immersive XR training systems for acupuncture education published between 2008 and 2026. AR systems were used only for acupoint localization, MR systems mainly for needling, and VR systems for localization, needling, and clinical reasoning. Most studies reported technical or usability outcomes, and only 7 of 26 studies used a comparative design, so the educational benefits of these systems remain preliminary.

This study specifically focuses on immersive XR technologies, excluding nonimmersive desktop-based simulations. Historically, numerous studies have explored computer-based virtual acupuncture systems using standard monitors with a mouse, a camera, or desktop haptic input [46-48]. While these desktop applications contributed to visualizing meridians and acupoints [49,50], they lack the spatial immersion required for a realistic “first-person” training experience. We prioritized immersive technologies because acupuncture training depends on an understanding of 3D spatial relationships between surface acupoints and internal anatomical structures, rather than 2D visual memorization [10]. A recent meta-analysis reported that VR-based education was associated with higher knowledge and skill scores than traditional methods [11].

The included literature showed different distributions of XR modalities across educational tasks; these patterns should not be interpreted as evidence of modality-specific superiority. AR studies were concentrated on surface localization, overlaying acupoint information onto the user’s real-world view without external markers [9,44], although this focus remained on surface-level navigation rather than complex procedural tasks (Figure 4), whereas the included VR and MR studies more often addressed needling depth, angle, or 3D anatomical relationships. By rendering skin semitransparent or displaying tissue layers during virtual needling [28,45], these systems allow students to intuitively grasp insertion depth through visual or haptic cues [10]. Across XR modalities, a further distinction exists between IVR and SIVR systems. While both meet this review’s criteria for 1:1 spatial mapping, full visual immersion applies primarily to IVR, whereas the SIVR systems in this review use large displays, stereoscopic monitors, touch tables, tablets, or a monocular head-worn display that keep the physical surroundings in view [4,28,36,42-44].

The gap map (Figure 4) highlights that while VR is a versatile platform, no MR study addressed clinical reasoning, representing an important direction for future research. At the same time, the apparent pedagogical advantages of specific XR modalities should be interpreted in light of the uneven methodological rigor across studies. Only 7 of the 26 included studies used a comparative or experimental design. Most instead reported technical outcomes, such as positional accuracy or usability, rather than direct measures of learning. This imbalance was most evident for AR, where none of the 5 studies used a comparative design. Given this concentration of noncomparative designs, claims about the relative pedagogical advantage of any single XR modality should be regarded as preliminary until confirmed by larger, adequately powered comparative trials. Nonetheless, the observed division of labor across modalities may reflect a practical distinction in task demands: AR’s concentration on acupoint localization addresses a primarily visual-spatial task, for which overlay-based guidance may be particularly useful, whereas VR and MR’s focus on needling depth and angle addresses a psychomotor task that typically requires repeated, feedback-driven practice to refine. This distribution also resembles reviews of head-mounted XR in surgical [12] and medical [13] education, which have mainly described procedural and anatomical training rather than higher-order clinical reasoning. Clinical reasoning instead requires integrative, case-based judgment that has traditionally been supported by nonimmersive virtual patient formats; the absence of MR studies in this domain may, therefore, reflect a weaker match between immersive spatial interfaces and the integrative, case-based nature of diagnostic reasoning, rather than an inherent technical shortfall of MR itself.

Immersive XR may also address two limitations of traditional training, safety and standardization. VR and MR may allow rehearsal of high-risk needling without needle insertion in human participants, potentially reducing exposure to iatrogenic injuries such as pneumothorax [5]. Unlike an earlier monitor-based system that overlaid acupoints on a camera image of the learner’s hand [47], immersive XR can capture quantitative data, including positional accuracy, insertion depth and angle, and procedure time, in 3D environments [10,30], which may support standardized evaluations such as the Objective Structured Clinical Examination, although safety and assessment outcomes were not evaluated in the included studies. In one comparative study, learners rated the VR system highly for interest and interactivity, although satisfaction was surveyed only in the VR group [28]. For educators, XR systems may offer a platform that helps bridge theoretical knowledge and clinical practice. These technologies may facilitate active engagement through 3D anatomical interaction and immediate feedback. Although these features are promising, implementation barriers remain, including high equipment costs [9] and the need to match XR content to learners’ prior anatomical knowledge [4]. Therefore, future efforts should focus on embedding XR within competency-based education to support standardized training and objective assessment.

### Limitations

Despite these advancements, several challenges remain. The most notable is the lack of realistic haptic feedback. Although several desktop-based systems have implemented force-feedback using mechanical haptic arms [51,52], integrating such sophisticated haptics into wireless, immersive HMD environments remains technically difficult. Most included systems relied on visual cues or basic vibrotactile responses [10,30], and only 2 used force-feedback devices [24,28]; these approaches cannot fully replicate the deqi sensation or tissue resistance found in physical simulators. Haptic interfaces compatible with head-mounted XR are needed to close this gap. Accessibility is another concern, as advanced markerless registration often requires expensive hardware like 3D scanners [9]. Moreover, most models are currently restricted to specific body parts; whole-body models would be needed to cover the full meridian system. Including conference proceedings captured recent XR developments, but their variable peer review may have admitted less rigorously validated systems, which would tend to overstate technical maturity. The 26 included studies were concentrated in East Asia (25/26), so the map may not cover XR training developed elsewhere or indexed in databases that were not searched, including Japanese databases such as J-STAGE. Finally, formal risk-of-bias appraisal was not performed, consistent with the mapping objective of a scoping review; the evidence tiers therefore describe study design and should not be interpreted as ratings of methodological quality. Objective technical performance metrics, such as localization and depth error margins, were synthesized to characterize technical maturity, but these metrics do not substitute for formal risk-of-bias appraisal. Classification of immersion depended on how authors described their hardware.

### Conclusions

This scoping review identified 26 studies that applied IVR, SIVR, AR, and MR training systems to acupuncture education, addressing acupoint localization (n=13), needling skills (n=11), and clinical reasoning (n=2). AR systems were used only for localization, MR systems mainly for needling, and VR systems across all three domains; no AR or MR study addressed clinical reasoning. Unlike earlier reviews of physical phantom models or of XR in general health professions education, this review maps immersive systems by modality, level of immersion, and educational task. Only 7 studies used a comparative design, and most reported technical or usability outcomes, so the educational benefits of these systems remain preliminary. Randomized trials comparing XR-based and conventional training on objective localization and needling outcomes, with retention follow-up, are needed before these systems are adopted for competency assessment. Trials comparing XR modalities on the same task would then show which modality suits which training objective.

## Supplementary material

10.2196/93369 Multimedia Appendix 1 Search strategy.

10.2196/93369 Checklist 1 PRISMA-ScR checklist.

10.2196/93369 Checklist 2 PRISMA-S checklist.

## Abbreviations

AR: augmented reality
HMD: head-mounted display
IVR: immersive virtual reality
MR: mixed reality
NIVR: nonimmersive virtual reality
OSCE: Objective Structured Clinical Examination
PCC: population, concept, context
PRISMA-S: Preferred Reporting Items for Systematic Reviews and Meta-Analyses Literature Search Extension
PRISMA-ScR: Preferred Reporting Items for Systematic Reviews and Meta-Analyses extension for Scoping Reviews
SIVR: semi-immersive virtual reality
VR: virtual reality
XR: extended reality

## References

[1] Park HS, Jeong HI, Sung SH, Kim KH (2023). Acupuncture treatment for hip pain: a systematic review and meta-analysis. Healthcare (Basel).

[2] Zhang Y (2021). Interpretation of acupoint location in traditional Chinese medicine teaching: implications for acupuncture in research and clinical practice. Anat Rec (Hoboken).

[3] Jang JE, Lee YS, Jang WS (2022). Trends in acupuncture training research: focus on practical phantom models. J Acupunct Res.

[4] Xu A, Li Z (2025). The challenges and solutions of acupuncture education in the context of new medicine: a case study of Beijing University of Chinese Medicine. Med Acupunct.

[5] Jeong H, Kim JH, Park H (2024). A case report on hemopneumothorax caused by acupuncture at Huatuo–Jiaji points. Heliyon.

[6] Cho E, Lee JH, Kwon OS, Hong J, Cho NG (2021). Assessment of acupuncture and moxibustion medicine clinical practice using the objective structured clinical examination. J Acupunct Res.

[7] Wilcha RJ (2020). Effectiveness of virtual medical teaching during the COVID-19 crisis: systematic review. JMIR Med Educ.

[8] Attardi SM, Harmon DJ, Barremkala M (2022). An analysis of anatomy education before and during COVID-19: August-December 2020. Anat Sci Educ.

[9] Chiou SY, Chang HH, Chen YC, Liu GH (2025). Augmented reality navigation for acupuncture procedures with smart glasses. Electronics (Basel).

[10] Guruge K, Herath HMKKMB, Madusanka N (2025). Advancing medical training with mixed reality and haptic feedback simulator for acupuncture needling. Sensors (Basel).

[11] Sung H, Kim M, Park J, Shin N, Han Y (2024). Effectiveness of virtual reality in healthcare education: systematic review and meta-analysis. Sustainability.

[12] Qi Z, Corr F, Grimm D, Nimsky C, Bopp MHA (2024). Extended reality-based head-mounted displays for surgical education: a ten-year systematic review. Bioengineering (Basel).

[13] Barteit S, Lanfermann L, Bärnighausen T, Neuhann F, Beiersmann C (2021). Augmented, mixed, and virtual reality-based head-mounted devices for medical education: systematic review. JMIR Serious Games.

[14] Tricco AC, Lillie E, Zarin W (2018). PRISMA extension for scoping reviews (PRISMA-ScR): checklist and explanation. Ann Intern Med.

[15] Arksey H, O’Malley L (2005). Scoping studies: towards a methodological framework. Int J Soc Res Methodol.

[16] Levac D, Colquhoun H, O’Brien KK (2010). Scoping studies: advancing the methodology. Implement Sci.

[17] Immersive XR technologies in acupuncture education: a scoping review protocol. Open Science Framework (OSF).

[18] Ware C, Arthur K, Booth KS Fish tank virtual reality.

[19] Lombard M, Ditton T (1997). At the heart of it all: the concept of presence. J Comput Mediat Commun.

[20] Cipresso P, Giglioli IAC, Raya MA, Riva G (2018). The past, present, and future of virtual and augmented reality research: a network and cluster analysis of the literature. Front Psychol.

[21] Rethlefsen ML, Kirtley S, Waffenschmidt S (2021). PRISMA-S: an extension to the PRISMA statement for reporting literature searches in systematic reviews. Syst Rev.

[22] Lefebvre C, Glanville J, Briscoe S, Higgins JPT, Thomas J, Chandler J, Cumpston M, Li T, Page MJ, Welch VA (2025). Cochrane Handbook for Systematic Reviews of Interventions Version.

[23] Jiang H, Zhang Y, Song A (2024). Application value of VR technology in traditional Chinese medicine clinical practice teaching [Article in Chinese]. Chin Med Mod Distance Educ China.

[24] Yan R, Hu X, Zhu C (2024). Design of an acupuncture treatment teaching system based on VR haptic interaction [Article in Chinese]. Comput Knowl Technol.

[25] Lu C, Ding S (2021). Development of a VR version of TCM twelve meridians teaching system based on UE4 [Article in Chinese]. Electron Technol Softw Eng.

[26] Gao H, Gao Y, Kan H, Chen G en Research on development and design of virtual acupuncture teaching software based on HTC VIVE.

[27] Chen X, Yang H, Ji Y, Chen D 3D real-time face acupoints recognition system based on HoloLens 2.

[28] Rao XD, Yu HB, Wu JH, Zhong WZ, Huang XX (2020). Practical experience of virtual acupuncture and moxibustion teaching system in the operation teaching course of Acupuncture Sciences. Zhongguo Zhen Jiu.

[29] Chen Y (2025). Exploration and evaluation of the application of virtual simulation technology in acupuncture training practice [Article in Chinese]. Educ Teach Forum.

[30] Sun Q, Huang J, Zhang H, Craig P, Yu L, Lim EG Design and development of a mixed reality acupuncture training system.

[31] Wu L, Chellappa V, Xie Q, Mao X, Luximon Y, Yu R (2026). Immersive virtual reality for acupuncture knowledge and practical skills: a comparative educational study. Front Public Health.

[32] Sun J, Liu J, Yang J (2023). Application of virtual reality technology in practical teaching of acupuncture, massage and cosmetology [Article in Chinese]. Chin J Aesthet Med.

[33] Chen L, Mao H, Zhang W (2022). Application of experiment project of virtual simulation in the teaching of acupuncture anesthesia [Article in Chinese]. Educ Chin Med.

[34] Zhang X, Jia C, Xing H (2022). Application and exploration of virtual reality acupuncture teaching system [Article in Chinese]. J Hebei Tradit Chin Med Pharmacol.

[35] Geng X, Hu F, Wang Z, Zhao M, Wang M (2019). Virtual acupuncture teaching practice assistant system based on Unity3D [Article in Chinese]. Keji Feng.

[36] Tong CKS, Chan OCM, Yu ECL (2023). The future of acupuncture education: 3D Augmented Reality (AR) acupuncture model for Chinese Medicine. HKIE Trans.

[37] Chen ZB, Fan ZG, Wang XC (2013). Research of the virtual acupuncture training system VAMT. Inf Technol J.

[38] Wang X, Yang Y, Zhou K MRUCT: mixed reality assistance for acupuncture guided by ultrasonic computed tomography.

[39] Zhang M, Zheng Y, Cui C, Meng Z, Own CM Preliminary application of gesture recognition to virtual acupuncture.

[40] Ryu CJ, Lee SD, Han SJ (2020). Design of acupuncture contents and dummy for acupuncture point training system [Article in Korean]. J Adv Eng Technol.

[41] Ryu CJ, Lee SD, Han SJ (2020). Design of acupuncture controller and dummy for acupuncture training system based on MR. Smart Media J.

[42] Yang S, Ryu C, Kim S, Kim J (2021). A development of an acupoints education table using 3D technology and augmented reality. Korean J Acupunct.

[43] Chiou SY, He MR (2024). Acupuncture navigation method integrated with augmented reality. Biomed Mater Eng.

[44] Jiang H, Starkman J, Kuo CH, Huang MC (2015). Acu glass: quantifying acupuncture therapy using Google Glass. EAI Endorsed Trans Perv Health Tech.

[45] Kanehira R, Shoda A, Yagihashi M, Narita H, Fujimoto H Development of an acupuncture training system using virtual reality technology.

[46] Heng PA, Wong TT, Yang R (2006). Intelligent inferencing and haptic simulation for Chinese acupuncture learning and training. IEEE Trans Inf Technol Biomed.

[47] Kim GH, Yu HB (2000). Hand acupuncture supporting system using mixed reality scheme [Article in Korean]. Trans Korea Inf Process Soc.

[48] Heng PA, Xie Y, Wang X, Chui YP, Wong TT (2006). Medicine Meets Virtual Reality 14 – Accelerating Change in Healthcare: Next Medical Toolkit, MMVR 2006.

[49] Zheng L, Qin B, Zhuang T, Tiede U, Höhne KH (2005). Localization of acupoints on a head based on a 3D virtual body. Image Vis Comput.

[50] Huang W, Yang Y, Yang L, Yan M (2020). Application of acupuncture acupoint visual teaching system by neural regulation. World Neurosurg.

[51] Cheng Z, Wang H sheng, Min Y jiang, Yan Z guo, Hong ZT, Zhuang T ge (2007). Preliminary study on force feedback of acupuncture in virtual reality based on the visible human. Zhongguo Yi Liao Qi Xie Za Zhi.

[52] Heng PA, Wong TT, Leung KM, Chui YP, Sun H A haptic needle manipulation simulator for Chinese acupuncture learning and training.

