# Supplementary material for: Application of Immersive and Semi-Immersive Extended Reality Training Systems in Acupuncture Education: Scoping Review
Source: JMIR Serious Games. 2026 Oct 21;14:e93369. doi: 10.2196/93369 (PMC13686450; doi:10.2196/93369)
Supplement: Supplementary file 1 [file games-v14-e93369-s002.pdf]

PRISMA-ScR Checklist

Manuscript #93369. Application of Immersive and Semi-Immersive Extended Reality (XR) Training Systems in Acupuncture Education: A Scoping Review

Section	#	Checklist item	Location(s) reported
**TITLE**			
Title	1	Identify the report as a scoping review.	Title: "... : A Scoping Review"
**ABSTRACT**			
Structured summary	2	Provide a structured summary that includes (as applicable) background, objectives, eligibility criteria, sources of evidence, charting methods, results, and conclusions that relate to the review questions and objectives.	Abstract (Background, Objective, Methods, Results, Conclusions, Registration)
**INTRODUCTION**			
Rationale	3	Describe the rationale for the review in the context of what is already known. Explain why the review questions/objectives lend themselves to a scoping review approach.	Introduction, paragraphs 1-3
Objectives	4	Provide an explicit statement of the questions and objectives being addressed with reference to their key elements (e.g., population or participants, concepts, and context) or other relevant key elements used to conceptualize the review questions and/or objectives.	Introduction, final paragraph (three research questions framed with PCC)
**METHODS**			
Protocol and registration	5	Indicate whether a review protocol exists; state if and where it can be accessed (e.g., a Web address); and if available, provide registration information, including the registration number.	Methods, Protocol and Registration; Abstract, Registration (OSF osf.io/pvu8e)
Eligibility criteria	6	Specify characteristics of the sources of evidence used as eligibility criteria (e.g., years considered, language, and publication status), and provide a rationale.	Methods, Eligibility Criteria
Information sources	7	Describe all information sources in the search (e.g., databases with dates of coverage and contact with authors to identify additional sources), as well as the date the most recent search was executed.	Methods, Information Sources
Search	8	Present the full electronic search strategy for at least 1 database, including any limits used, such that it could be repeated.	Methods, Search; full electronic search strategies for all eight databases in Multimedia Appendix 1
Selection of sources of evidence	9	State the process for selecting sources of evidence (i.e., screening and eligibility) included in the scoping review.	Methods, Selection of Sources of Evidence
Data charting process	10	Describe the methods of charting data from the included sources of evidence (e.g., calibrated forms or forms that have been tested by the team before their use, and whether data charting was done independently or in duplicate) and any processes for obtaining and confirming data from investigators.	Methods, Data Charting Process
Data items	11	List and define all variables for which data were sought and any assumptions and simplifications made.	Methods, Data Items
Critical appraisal of individual sources of evidence	12	If done, provide a rationale for conducting a critical appraisal of included sources of evidence; describe the methods used and how this information was used in any data synthesis (if appropriate).	Methods, Critical Appraisal of Individual Sources of Evidence. Not performed; rationale stated.
Synthesis of results	13	Describe the methods of handling and summarizing the data that were charted.	Methods, Synthesis of Results
**RESULTS**			
Selection of sources of evidence	14	Give numbers of sources of evidence screened, assessed for eligibility, and included in the review, with reasons for exclusions at each stage, ideally using a flow diagram.	Results, Selection of Sources of Evidence; Figure 1 (PRISMA flow diagram)
Characteristics of sources of evidence	15	For each source of evidence, present characteristics for which data were charted and provide the citations.	Results, Characteristics of Sources of Evidence; Table 1; Figure 2
Critical appraisal within sources of evidence	16	If done, present data on critical appraisal of included sources of evidence (see item 12).	Not applicable. Critical appraisal was not performed, as stated in Methods, Critical Appraisal of Individual Sources

			of Evidence.
Results of individual sources of evidence	17	For each included source of evidence, present the relevant data that were charted that relate to the review questions and objectives.	Results, Results of Individual Sources of Evidence; Table 1
Synthesis of results	18	Summarize and/or present the charting results as they relate to the review questions and objectives.	Results, Synthesis of Results; Figure 3 (evidence gap map)
**DISCUSSION**			
Summary of evidence	19	Summarize the main results (including an overview of concepts, themes, and types of evidence available), link to the review questions and objectives, and consider the relevance to key groups.	Discussion, Summary of Evidence
Limitations	20	Discuss the limitations of the scoping review process.	Discussion, Limitations
Conclusions	21	Provide a general interpretation of the results with respect to the review questions and objectives, as well as potential implications and/or next steps.	Conclusions
**FUNDING**			
Funding	22	Describe sources of funding for the included sources of evidence, as well as sources of funding for the scoping review. Describe the role of the funders of the scoping review.	Funding statement following the Conclusions. Funding for individual included sources of evidence was not charted.

Source: Tricco AC, Lillie E, Zarin W, et al. PRISMA Extension for Scoping Reviews (PRISMA-ScR): Checklist and Explanation. Ann Intern Med. 2018;169(7):467-473.
