# Supplementary material for: Application of Immersive and Semi-Immersive Extended Reality Training Systems in Acupuncture Education: Scoping Review
Source: JMIR Serious Games. 2026 Oct 21;14:e93369. doi: 10.2196/93369 (PMC13686450; doi:10.2196/93369)
Supplement: Supplementary file 2 [file games-v14-e93369-s001.docx]

**Multimedia Appendix 1. List of Terms Used and Search Results**

| **No.** | **Database** | **Search No.** | **Terms** | **Search result** |
| --- | --- | --- | --- | --- |
| 1 | PubMed (pubmed.ncbi.nlm.nih.gov) | #1 | "Acupuncture"[Mesh] OR "Acupuncture Therapy"[Mesh] OR "Acupuncture Points"[Mesh] OR "Acupuncture Analgesia"[Mesh] OR "Electroacupuncture"[Mesh] OR "Moxibustion"[Mesh] OR "Meridians"[Mesh] OR "Medicine, East Asian Traditional"[Mesh] OR "Medicine, Korean Traditional"[Mesh] OR "Medicine, Chinese Traditional"[Mesh] OR "Dry Needling"[Mesh] OR acupunctur*[tiab] OR electroacupunctur*[tiab] OR acupoint*[tiab] OR "acupuncture point"[tiab] OR "acupuncture points"[tiab] OR meridian*[tiab] OR moxibust*[tiab] OR needling[tiab] OR "needle insertion"[tiab] OR "needle manipulation"[tiab] OR "dry needling"[tiab] OR acupressure[tiab] OR pharmacopunctur*[tiab] OR "auricular acupuncture"[tiab] OR "auricular therapy"[tiab] OR "scalp acupuncture"[tiab] OR "Korean medicine"[tiab] OR "Korean traditional medicine"[tiab] OR "Oriental medicine"[tiab] OR "East Asian medicine"[tiab] OR "Chinese medicine"[tiab] OR "traditional Chinese medicine"[tiab] OR TCM[tiab] OR Kampo[tiab] OR "de qi"[tiab] OR deqi[tiab] | 133,619 |
|  |  | #2 | "Virtual Reality"[Mesh] OR "Augmented Reality"[Mesh] OR "Virtual Reality Exposure Therapy"[Mesh] OR "Holography"[Mesh] OR "virtual realit*"[tiab] OR "augmented realit*"[tiab] OR "mixed realit*"[tiab] OR "extended realit*"[tiab] OR "virtual environment*"[tiab] OR "virtual simulat*"[tiab] OR immersiv*[tiab] OR metavers*[tiab] OR haptic*[tiab] OR "force feedback"[tiab] OR "tactile feedback"[tiab] OR "head-mounted display*"[tiab] OR "head mounted display*"[tiab] OR HMD[tiab] OR VR[tiab] OR XR[tiab] OR "serious game*"[tiab] OR gamif*[tiab] OR "3D simulat*"[tiab] OR "three-dimensional simulat*"[tiab] OR stereoscop*[tiab] OR "360-degree video"[tiab] OR "360 degree video"[tiab] OR "digital twin*"[tiab] OR hologra*[tiab] OR Oculus[tiab] OR "Meta Quest"[tiab] OR HoloLens[tiab] OR Vive[tiab] OR "Google Cardboard"[tiab] OR "Leap Motion"[tiab] OR ("AR"[ti] AND (realit*[tiab] OR immersiv*[tiab] OR overlay*[tiab])) OR ("MR"[ti] AND (realit*[tiab] OR immersiv*[tiab])) | 101,457 |
|  |  | #3 | #1 AND #2 | 548 |
| 2 | Embase (embase.com, advanced search) | #1 | 'acupuncture'/exp OR 'electroacupuncture'/exp OR 'moxibustion'/exp OR 'acupuncture point'/exp OR 'meridian'/exp OR 'dry needling'/exp OR 'traditional Chinese medicine'/exp OR 'Korean traditional medicine'/exp OR 'traditional medicine'/exp OR acupunctur*:ti,ab,kw OR electroacupunctur*:ti,ab,kw OR acupoint*:ti,ab,kw OR meridian*:ti,ab,kw OR moxibust*:ti,ab,kw OR needling:ti,ab,kw OR 'needle insertion':ti,ab,kw OR 'needle manipulation':ti,ab,kw OR 'dry needling':ti,ab,kw OR acupressure:ti,ab,kw OR pharmacopunctur*:ti,ab,kw OR 'auricular acupuncture':ti,ab,kw OR 'scalp acupuncture':ti,ab,kw OR 'Korean medicine':ti,ab,kw OR 'Oriental medicine':ti,ab,kw OR 'East Asian medicine':ti,ab,kw OR 'Chinese medicine':ti,ab,kw OR 'traditional Chinese medicine':ti,ab,kw OR kampo:ti,ab,kw OR deqi:ti,ab,kw | 293,414 |
|  |  | #2 | 'virtual reality'/exp OR 'augmented reality'/exp OR 'mixed reality'/exp OR 'virtual reality exposure therapy'/exp OR 'serious game'/exp OR 'virtual reality':ti,ab,kw OR 'augmented reality':ti,ab,kw OR 'mixed reality':ti,ab,kw OR 'extended reality':ti,ab,kw OR 'virtual environment':ti,ab,kw OR 'virtual simulation':ti,ab,kw OR immersiv*:ti,ab,kw OR metavers*:ti,ab,kw OR haptic*:ti,ab,kw OR 'force feedback':ti,ab,kw OR 'tactile feedback':ti,ab,kw OR 'head mounted display':ti,ab,kw OR 'head-mounted display':ti,ab,kw OR hmd:ti,ab,kw OR vr:ti,ab,kw OR xr:ti,ab,kw OR 'serious game':ti,ab,kw OR gamif*:ti,ab,kw OR '3d simulation':ti,ab,kw OR stereoscop*:ti,ab,kw OR '360 degree video':ti,ab,kw OR 'digital twin':ti,ab,kw OR hologra*:ti,ab,kw OR oculus:ti,ab,kw OR 'meta quest':ti,ab,kw OR hololens:ti,ab,kw OR vive:ti,ab,kw OR 'google cardboard':ti,ab,kw OR 'leap motion':ti,ab,kw | 130,442 |
|  |  | #3 | #1 AND #2 | 1576 |
| 3 | Scopus (advanced document search) | #1 | TITLE-ABS-KEY(acupunctur* OR electroacupunctur* OR acupoint* OR meridian* OR moxibust* OR needling OR "needle insertion" OR "dry needling" OR acupressure OR pharmacopunctur* OR "auricular acupuncture" OR "scalp acupuncture" OR "Korean medicine" OR "Oriental medicine" OR "East Asian medicine" OR "Chinese medicine" OR "traditional Chinese medicine" OR Kampo OR deqi) | 220,932 |
|  |  | #2 | TITLE-ABS-KEY("virtual realit*" OR "augmented realit*" OR "mixed realit*" OR "extended realit*" OR "virtual environment*" OR immersiv* OR metavers* OR haptic* OR "force feedback" OR "head-mounted display*" OR HMD OR "serious game*" OR gamif* OR "3D simulat*" OR stereoscop* OR "360-degree video" OR "digital twin*" OR hologra* OR Oculus OR "Meta Quest" OR HoloLens OR Vive OR "Leap Motion") | 647,668 |
|  |  | #3 | #1 AND #2 | 1456 |
| 4 | IEEE Xplore (command search, All Metadata) | #1 | ("All Metadata":acupunctur* OR "All Metadata":acupoint* OR "All Metadata":moxibust* OR "All Metadata":meridian OR "All Metadata":needling OR "All Metadata":acupressure OR "All Metadata":"Korean medicine" OR "All Metadata":"Oriental medicine" OR "All Metadata":"traditional Chinese medicine" OR "All Metadata":"East Asian medicine") | 18,202 |
|  |  | #2 | ("All Metadata":"virtual reality" OR "All Metadata":"augmented reality" OR "All Metadata":"mixed reality" OR "All Metadata":"extended reality" OR "All Metadata":"virtual environment*" OR "All Metadata":immersive OR "All Metadata":metaverse OR "All Metadata":haptic* OR "All Metadata":"head-mounted display*" OR "All Metadata":HMD OR "All Metadata":"serious game*" OR "All Metadata":hologra* OR "All Metadata":"digital twin*") | 146,913 |
|  |  | #3 | #1 AND #2 | 798 |
| 5 | CNKI (oversea.cnki.net, professional search) | #1 | SU=('针灸'+'针刺'+'艾灸'+'灸法'+'穴位'+'腧穴'+'经络'+'电针'+'耳针'+'头针'+'针灸学'+'刺法灸法'+'毫针'+'韩医'+'韩医学') | 284,267 |
|  |  | #2 | SU=('虚拟现实'+'增强现实'+'混合现实'+'扩展现实'+'元宇宙'+'沉浸式'+'头戴式显示器'+'头显'+'力反馈'+'触觉反馈'+'数字孪生'+'严肃游戏'+'VR') | 83,251 |
|  |  | #3 | #1 AND #2 | 855 |
| 6 | RISS (riss.kr, advanced search, all fields) | #1 | 침구\|침술\|침치료\|"침 치료"\|침시술\|자침\|경혈\|경락\|한의학\|한방\|acupuncture\|acupoint\|"Korean medicine"\|meridian\|needling | 56,147 |
|  |  | #2 | 가상현실\|"가상 현실"\|증강현실\|"증강 현실"\|혼합현실\|확장현실\|메타버스\|햅틱\|시뮬레이터\|"virtual reality"\|"augmented reality"\|"mixed reality"\|metaverse\|haptic | 32,199 |
|  |  | #3 | #1 AND #2 | 450 |
| 7 | ScienceOn (scienceon.kisti.re.kr, advanced search, papers) | #1 | 침구\|침술\|침치료\|"침 치료"\|침시술\|자침\|경혈\|경락\|한의학\|한방\|acupuncture\|acupoint\|"Korean medicine"\|meridian\|needling | 155,074 |
|  |  | #2 | 가상현실\|"가상 현실"\|증강현실\|"증강 현실"\|혼합현실\|확장현실\|메타버스\|햅틱\|시뮬레이터\|"virtual reality"\|"augmented reality"\|"mixed reality"\|metaverse\|haptic | 223,048 |
|  |  | #3 | #1 AND #2 | 476 |
| 8 | OASIS (oasis.kiom.re.kr, integrated search) | #1 | 가상현실\|"가상 현실"\|증강현실\|"증강 현실"\|혼합현실\|확장현실\|메타버스\|햅틱\|시뮬레이터\|"virtual reality"\|"augmented reality"\|"mixed reality"\|metaverse\|haptic | 17 |
