# Supplementary material for: Application of Immersive and Semi-Immersive Extended Reality Training Systems in Acupuncture Education: Scoping Review
Source: JMIR Serious Games. 2026 Oct 21;14:e93369. doi: 10.2196/93369 (PMC13686450; doi:10.2196/93369)
Supplement: Supplementary file 3 [file games-v14-e93369-s003.docx]

**PRISMA-S Checklist**

Application of Immersive and Semi-Immersive Extended Reality Training Systems in Acupuncture Education: Scoping Review (JMIR Serious Games, manuscript 93369)

| **Section/topic** | **#** | **Checklist item** | **Location(s) reported** |
| --- | --- | --- | --- |
| **INFORMATION SOURCES AND METHODS** | | | |
| Database name | 1 | Name each individual database searched, stating the platform for each. | PubMed (National Library of Medicine interface), Embase, Scopus, IEEE Xplore, CNKI (overseas platform), RISS, ScienceOn, and OASIS, each searched on its own platform. Methods, Information Sources. |
| Multi-database searching | 2 | If databases were searched simultaneously on a single platform, state the name of the platform, listing all of the databases searched. | Not applicable. Each database was searched separately on its own platform. Methods, Information Sources. |
| Study registries | 3 | List any study registries searched. | Not applicable. No study registries were searched; trial registry records were outside the eligibility criteria. Methods, Eligibility Criteria. |
| Online resources and browsing | 4 | Describe any online or print source purposefully searched or browsed (eg, tables of contents, print conference proceedings, web sites), and how this was done. | Not applicable. No online or print sources were browsed beyond the database and citation searches. |
| Citation searching | 5 | Indicate whether cited references or citing references were examined, and describe any methods used for locating cited/citing references (eg, browsing reference lists, using a citation index, setting up email alerts for references citing included studies). | Backward and forward citation searching of the included studies supplemented the database search. Methods, Information Sources. Citation searching identified 647 records, of which 1 study was included. Results, Selection of Sources of Evidence; Figure 1. |
| Contacts | 6 | Indicate whether additional studies or data were sought by contacting authors, experts, manufacturers, or others. | Not applicable. No authors, experts, or manufacturers were contacted. |
| Other methods | 7 | Describe any additional information sources or search methods used. | Not applicable. No other information sources or search methods were used. |
| **SEARCH STRATEGIES** | | | |
| Full search strategies | 8 | Include the search strategies for each database and information source, copied and pasted exactly as run. | The search strategy for each database, copied exactly as run, with the number of records retrieved at each step. Multimedia Appendix 1; Methods, Search. |
| Limits and restrictions | 9 | Specify that no limits were used, or describe any limits or restrictions applied to a search (eg, date or time period, language, study design) and provide justification for their use. | No language, date, or study design limits were applied. Database document type filters were used to remove ineligible document types (1171 records, including 106 Embase trial registry records), consistent with the eligibility criteria. Methods, Search; Results, Selection of Sources of Evidence. |
| Search filters | 10 | Indicate whether published search filters were used (as originally designed or modified), and if so, cite the filter(s) used. | Not applicable. No published search filters were used or adapted. Methods, Search. |
| Prior work | 11 | Indicate when search strategies from other literature reviews were adapted or reused for a substantive part or all of the search, citing the previous review(s). | Not applicable. No search strategy from a previous review was reused. Methods, Search. |
| Updates | 12 | Report the methods used to update the search(es) (eg, rerunning searches, email alerts). | Not applicable. The searches were not rerun or updated. Methods, Search. |
| Dates of searches | 13 | For each search strategy, provide the date when the last search occurred. | All database searches were run on September 19, 2026. Methods, Information Sources; Multimedia Appendix 1. |
| **PEER REVIEW** | | | |
| Peer review | 14 | Describe any search peer review process. | The search strategies were internally reviewed by the review team before the searches were run; no formal external peer review was performed. Methods, Search. |
| **MANAGING RECORDS** | | | |
| Total records | 15 | Document the total number of records identified from each database and other information sources. | 6176 records from databases (PubMed, n=548; Embase, n=1576; Scopus, n=1456; IEEE Xplore, n=798; CNKI, n=855; RISS, n=450; ScienceOn, n=476; OASIS, n=17) and 647 records from citation searching. Results, Selection of Sources of Evidence; Figure 1; Multimedia Appendix 1. |
| Deduplication | 16 | Describe the processes and any software used to deduplicate records from multiple database searches and other information sources. | Records were deduplicated in Microsoft Excel by matching digital object identifiers and normalized titles within a publication-year window of 2 years; 1627 duplicates were removed before screening and 2 further duplicates were identified during screening. Methods, Selection of Sources of Evidence; Results, Selection of Sources of Evidence. |

From: Rethlefsen ML, Kirtley S, Waffenschmidt S, Ayala AP, Moher D, Page MJ, Koffel JB, PRISMA-S Group. PRISMA-S: an extension to the PRISMA statement for reporting literature searches in systematic reviews. Syst Rev. 2021;10(1):39.
